# Immediate effects of kinesiology taping on proprioception and functional performance in collegiate athletes with chronic ankle instability: evidence from event-related potentials

**DOI:** 10.1186/s13102-025-01468-w

**Published:** 2025-12-12

**Authors:** Cheng-Liang Chang, Han-Yun Lin, Yu-Ting Tseng, Chien-Yu Pan, Fu-Chen Chen, Chia-Liang Tsai

**Affiliations:** 1 Lab of Cognitive Neurophysiology, Institute of Physical Education, Health and Leisure Studies, National Cheng Kung University, NO. 1, University Road, Tainan, 701 Taiwan; 2 Department of Kinesiology, National Tsing Hua University, Hsinchu , Taiwan; 3 Department of Physical Education, National Kaohsiung Normal University, Kaohsiung , Taiwan; 4 Department of Psychology, National Cheng Kung University, Tainan, Taiwan

**Keywords:** Kinesiology taping, Chronic ankle instability, Proprioception, Functional performance, Event-Related potentials

## Abstract

**Background:**

Chronic ankle instability (CAI) is associated with proprioceptive deficits and impaired functional performance, predisposing athletes to recurrent injury. Kinesiology taping (KT) is widely applied in sports medicine; however, its neurophysiological effects on proprioception remain unclear, particularly at different tension levels.

**Objective:**

To determine the immediate effects of KT tension on proprioception, functional performance, and cortical processing in collegiate athletes with CAI.

**Methods:**

Thirty athletes with CAI completed two experimental sessions (35% tension KT and 0% tension KT) in a within-subject crossover design. Proprioception was evaluated with a robotic ankle system using just noticeable difference (JND) and interval of uncertainty (IU), while simultaneous EEG captured event-related potentials (ERPs). Functional performance was evaluated using single-leg stance (SLS), single-leg hop (SLH), and single-leg lateral hop (SLLH) tests. Repeated-measures ANOVAs and correlation analyses were performed.

**Results:**

Application of 35% KT significantly reduced JND, whereas 0% KT increased JND. IU showed no significant changes. Functional outcomes improved under 35% KT for SLS (eyes closed) and SLLH-success, whereas SLLH-errors increased following 0% KT. ERPs analysis revealed stable N1 amplitude with 35% KT but significant reductions with 0% KT. Correlation analysis showed that decreases in JND were positively associated with stronger N1 responses, whereas both JND and IU were significantly related to SLLH performance.

**Conclusion:**

KT with appropriate tension immediately enhances proprioceptive acuity, cortical responsiveness, and functional stability in athletes with CAI, whereas placebo taping may disrupt sensory processing and impair performance. Electrophysiological measures provide valuable mechanistic insights for optimizing KT application in clinical and sports settings.

## Introduction

Ankle sprain is one of the most common sports-related injuries among college athletes. It accounts for more than 15% of all reported injuries [1]. Approximately 40% of athletes who experience an initial ankle sprain develop chronic ankle instability (CAI) [2]. According to the International Ankle Consortium, CAI is defined as a condition in which individuals experience recurrent ankle sprains and/or persistent symptoms, such as pain, perceived instability, weakness, and swelling, following a significant ankle sprain [3]. This condition is particularly common in court-based sports such as soccer, basketball, and volleyball, where frequent cutting, jumping, and landing maneuvers place high demands on ankle stability [4].

The pathophysiology of CAI involves not only mechanical insufficiencies, such as ligament laxity, restricted range of motion, and joint malalignment, but also sensorimotor deficits [2]. An important element of sensorimotor impairment is proprioceptive dysfunction, which is defined as the reduced ability to detect joint position and movement [2, 5, 6]. Intact proprioception is necessary for the neuromuscular control system to maintain stability in a dynamic environment. In contrast, inadequate proprioceptive feedback may contribute to delayed muscle activation, inaccurate joint positioning, and disrupted movement coordination, all of which increase the risk of re-injury [7–9]. Individuals with CAI frequently demonstrate reduced functional performance in tasks, such as single-leg hopping, balance control, and agility drills, which further limits their athletic participation and increases the risk of subsequent injury. These single-limb tasks place greater demands on proprioceptive feedback and neuromuscular control than double-limb movements, as they require continuous integration of somatosensory information to maintain joint stability and postural alignment, thereby providing a more sensitive assessment of proprioceptive deficits on the affected side [5, 10–13].

Because of the multifaceted nature of CAI, enhancing proprioceptive input and neuromuscular responsiveness is essential for injury prevention and effective rehabilitation [6, 14]. The immediate modulation of proprioceptive processing may lead to a meaningful improvement in functional and sports performance, which are critical for athletes. Of the various therapeutic approaches, kinesiology taping (KT) has garnered attention as a potential strategy to influence proprioceptive and neuromuscular function in individuals with CAI [7, 15–18]. KT is widely used in sports and rehabilitation to enhance circulation, reduce swelling, relieve pain, and improve neuromuscular control [19]. Unlike rigid athletic tape (AT), KT is elastic and designed to mimic the properties of human skin, thus allowing a full range of motion while providing continuous tactile stimulation. Thus, KT offers a unique approach in which variations in tension can produce distinct physiological and functional outcomes.

The proprioceptive effects of KT may arise from stimulation of cutaneous mechanoreceptors, which can enhance afferent feedback to the central nervous system, thus influencing proprioceptive acuity. By applying controlled tension to the skin over targeted muscles or joints, KT can induce localized traction and shear forces, which increase sensory input thereby facilitating more accurate motor control [20–22]. For individuals with CAI, the effects of KT on ankle proprioception and function remain unknown. For individuals with CAI, application of KT has been reported to produce beneficial effects, such as improvements in single-limb hurdle speed and vertical jump performance without affecting heel-rise performance [15], a decrease in force sense deficits following a 72-hour application [16], or enhanced ankle inversion proprioceptive performance during landing [7]. Conversely, no significant influence on balance control, muscle activation, or ankle kinesthesia has been observed in another study [17]. One possible source of these discrepancies is the variation in KT tension applied to study subjects, which generally ranges from 0% to 35% [15, 17, 23]. Because mechanoreceptor activation depends on the amount of skin stretch and pressure induced, differences in tension may affect the extent to which KT enhances, disrupts, or fails to alter proprioceptive input. Therefore, it is unclear whether KT improves proprioceptive acuity, and no studies have examined this process using direct neurophysiological measurements that can reveal how proprioceptive information is processed at the cortical level.

In the present study, we examined the effects of KT using psychophysical and neurophysiological measures. Determining KT efficacy requires a precise assessment of proprioception, which reflects the integrity of sensorimotor processing underlying joint stability and motor control. Among psychophysical approaches, joint motion sense tasks that quantify the just noticeable difference (JND) and interval of uncertainty (IU) provide reliable indices of proprioceptive sensitivity and perceptual precision [24]. JND reflects the smallest perceivable difference between two joint movements, whereas IU represents the variability and consistency of perceptual judgments. Although JND and IU have not been specifically examined in CAI, they have been shown to sensitively detect proprioceptive deficits in other musculoskeletal and neurological conditions, supporting their applicability in this context [25, 26]. Importantly, JND and IU are derived using the method of constant stimuli, where paired stimuli are presented in randomized order. This prevents participants from anticipating movement onset or direction, thereby reducing expectancy bias and errors associated with reaction time and improving the accuracy of proprioceptive discrimination. The assessment of JND and IU occurs immediately after each passive movement trial, under standardized conditions of movement intensity and execution velocity (e.g., 15°/s), ensuring consistency in testing context. This contrasts with the threshold to detection of passive motion (TTDPM), which relies on predictable ascending or descending stimulus sequences and is therefore more susceptible to anticipatory responses, particularly in individuals with residual proprioceptive function or extensive sport-related movement experience [27].

Although these assessments can capture psychophysical aspects of proprioception, they do not reveal how proprioceptive input is processed at the cortical level. Therefore, neurophysiological methods, such as electroencephalography (EEG) and event-related potentials (ERPs), have been used to examine the cortical dynamics of sensorimotor integration. ERPs provide temporally precise indices of early cortical responses to proprioceptive stimulation. The N1 component, typically observed at 100–150 ms after stimulus onset, may reflect early sensory processing and attention engagement. Studies of diverse populations support the sensitivity of the N1 component to proprioceptive integrity. For example, Tseng et al. [28] found that children with developmental coordination disorder show reduced N1 amplitudes during joint position sense tasks indicating reduced proprioceptive afferent inflow. Similarly, Toledo et al. [29] observed attenuated N1 amplitudes and prolonged N1 latencies in older adults relative to young adults, indicating an age-related reduction in the efficiency of proprioceptive processing. Although N1 characteristics have not been directly examined in individuals with CAI, previous EEG studies have demonstrated altered cortical activity related to sensorimotor control in this population. For instance, Needle et al. [30] reported reduced somatosensory evoked potentials and decreased sensorimotor cortical excitability in individuals with CAI, suggesting impaired afferent integration and cortical modulation. These findings imply that proprioceptive deficits in CAI may be accompanied by diminished cortical responsiveness to sensory input, which is analogous to the reduced or delayed N1 responses observed in populations with impaired proprioception. Therefore, interventions, such as KT, which enhance cutaneous and joint mechanoreceptor input, may modulate cortical processing of proprioceptive information and result in measurable changes in N1 amplitude or latency. This rationale supports examining both psychophysical measures of proprioception and N1 indices to assess the immediate effects of KT in athletes with CAI.

The role of KT in CAI remains unclear. Although several studies have examined proprioceptive changes following KT application in this population [7, 15–17], none have combined electrophysiological assessments with precise proprioception measurements and functional performance. Therefore, the primary aim of this study was to clarify the different effects of KT tensions on proprioception and to investigate the underlying neural mechanisms using electrophysiological measures. Furthermore, this study sought to determine whether changes in functional performance are associated with alterations in proprioceptive acuity or cortical processing. Such findings would provide valuable insight into the therapeutic potential of KT for individuals with CAI and help bridge the gap between neurophysiological modulation and practical performance outcomes. Moreover, establishing a relationship between proprioceptive function, neural activity, and functional performance can inform targeted rehabilitation strategies and evidence-based return-to-play decisions, which would ensure that athletes with CAI not only regain stability, but also achieve optimal performance capacity. Based on prior evidence, we hypothesize that (1) KT applied at 35% tension enhances proprioception and improves functional performance; (2) KT applied at 0% tension produces no significant changes; and (3) proprioceptive measures are significantly associated with functional performance outcomes and cortical electrophysiological indices.

## Materials and methods

### Participants

Thirty college athletes from open-skilled sports, such as basketball, volleyball, badminton, and soccer, were recruited for this study. The inclusion criteria, which were based on the guidelines of [31] were as follows: (a) age 18–25 years; (b) a self-reported history of at least one significant ankle sprain; (c) the initial sprain occurring at least 12 months before enrollment; (d) a history of the previously injured ankle “giving way,” recurrent sprain, or a sense of instability; and (e) a Cumberland Ankle Instability Tool (CAIT) score of 24 or lower. The exclusion criteria included: (a) a history of surgery to the musculoskeletal structures in either of the lower extremities; (b) a history of fracture in either lower extremity; (c) acute injury to the musculoskeletal structures of other lower-extremity joints within the past three months; and (d) allergy to KT. All subjects provided written informed consent before participation. The study protocol was approved by the Institutional Ethics Committee of National Cheng Kung University (Approval Number: 113–343-2), and all procedures conformed to the Declaration of Helsinki.

### Experimental procedure

This was a within-subject crossover design study. The subjects were asked to visit our acoustically shielded laboratory, which was maintained under controlled lighting and a temperature of 23–25 °C. During the first visit, demographic characteristics were recorded, and a CAIT questionnaire was administered. For each session, baseline measurements of proprioception and functional performance were conducted concomitantly with EEG recordings. KT was applied to the participants at 35% tension or 0% tension. Post-intervention assessments, which were identical to baseline measurements, were conducted immediately after application. All subjects underwent the 35% tension KT conditions first, followed by the 0% tension KT conditions, with a 2-month washout period between sessions. Although each KT condition in this study included its own pre- and post-intervention assessments, which allowed within-condition change scores to be compared without relying on baseline equivalence across conditions [32], the washout period was implemented in the non-randomized KT order to minimize potential carryover effects and strengthen the validity of the between-condition comparison [33]. In addition, to minimize circadian effects, each participant was scheduled to attend both sessions at approximately the same time of day.

### KT intervention

**Fig. 1:**
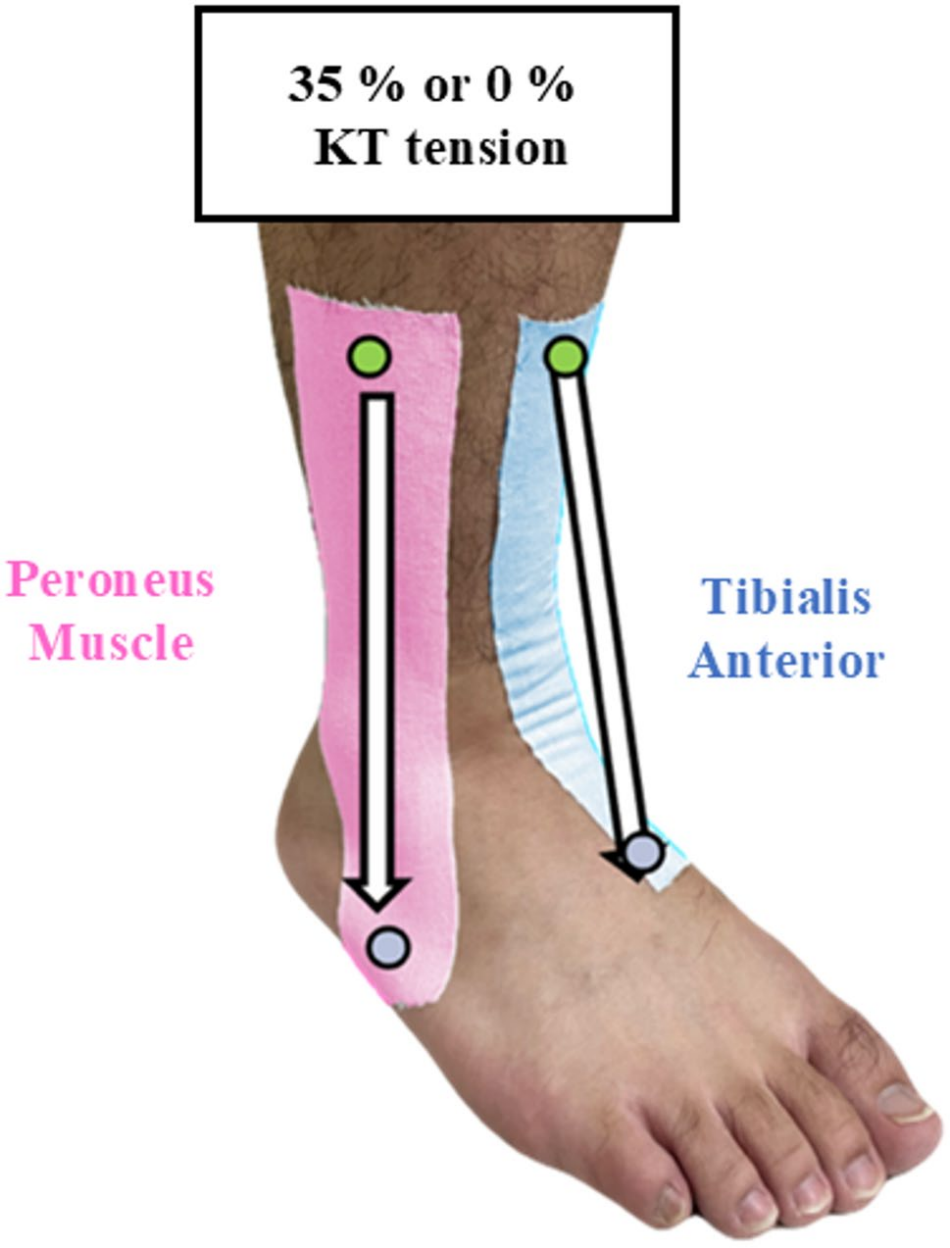
Kinesiology taping (KT) technique. Two I-band KT tapes were applied over the tibialis anterior (blue) and peroneus muscle groups (pink) using a facilitatory technique

As shown in Fig. 1, two I-band KT strips (SPORTSTEX, Korea) were applied to the skin over the tibialis anterior (blue) and peroneus muscle groups (pink) of each subject under either condition. These muscles were selected because they are involved in ankle stability and proprioceptive control in individuals with CAI [34]. A facilitatory KT application was used, which was applied from origin to insertion, as this approach effectively modulates activation of the targeted muscle groups and enhances proprioception [35, 36]. For the tibialis anterior, the anchor was placed on the medial side above the ankle joint. The tape was extended to the dorsal aspect at the base of the first metatarsal bone. For the peroneal muscles, the anchor was placed on the lateral side above the ankle joint, and the tape was extended to the plantar aspect at the base of the fifth metatarsal bone. During the taping process, subjects wore an eye mask to ensure blinding of the application procedure. All KT applications were performed by the same physical therapist for procedural consistency. The tape length was calculated using the following formula:

*Taping cutting length = [(actual length − 8 cm/factor) + 8 cm] × 1.10*,

where the factor was set to 1.00 for the 0% tension condition and 1.35 for the 35% tension condition [37].

### Assessment tools for proprioception

To measure the psychophysical response to proprioceptive stimuli, we used a customized robotic ankle proprioception assessment system, which consisted of a plastic footplate and a control cabinet [28]. The footplate was operated by a servo system, which consisted of servo drives (MBDLT25SF, Panasonic) and servo motors (HMF042L1U2M, Panasonic), offering a precision of 4.29–5°. The signals were continuously integrated into the control program at a 200 Hz sampling rate. The system generated movement speeds from 0.01°/s to 30°/s, which were adjustable in increments of 0.01°/s.

**Fig. 2:**
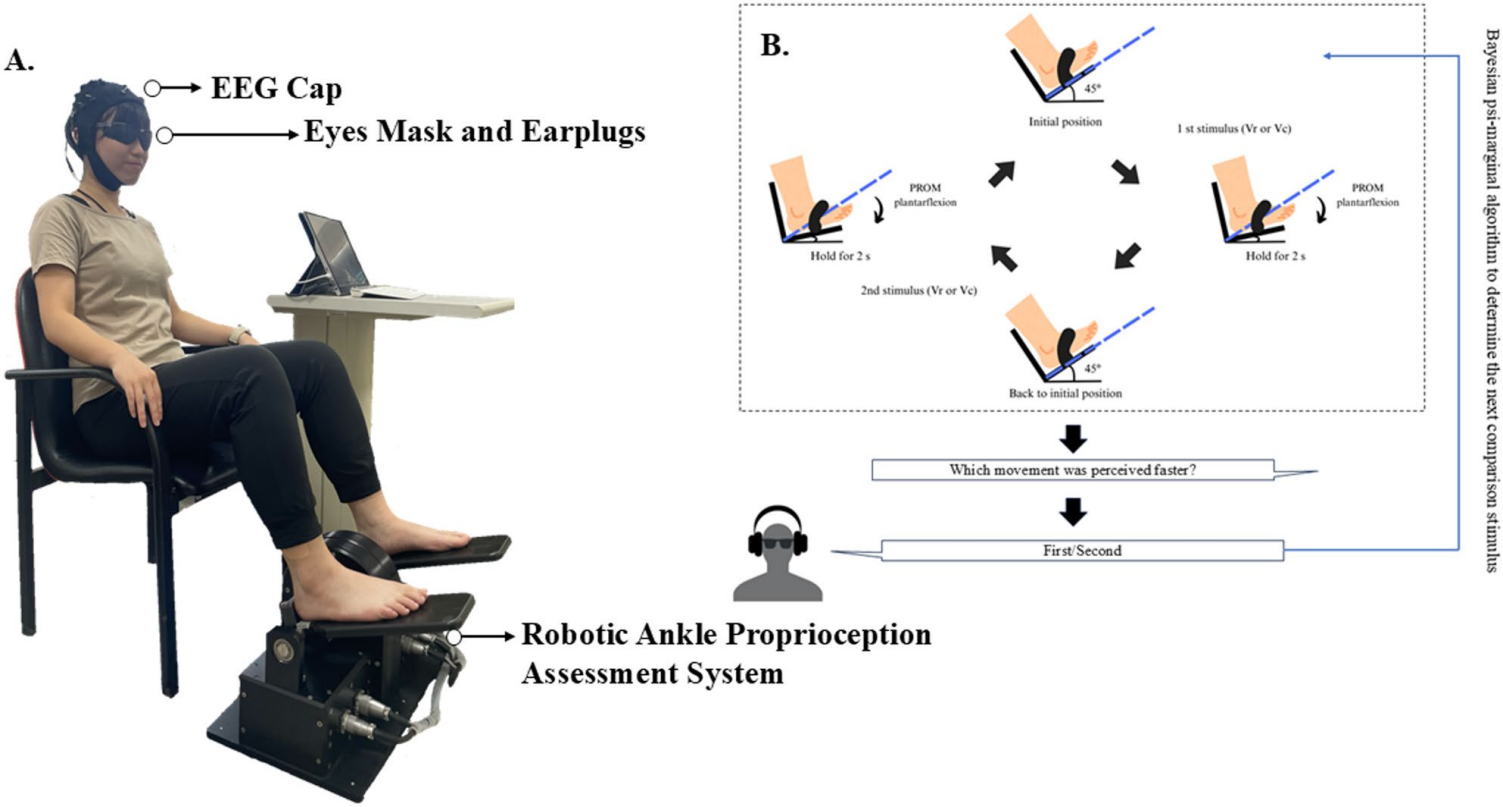
Experiment setup and procedure.** A** Robotic ankle proprioception system with a participant wearing an EEG cap, eye mask, and earplugs. **B** In each trial, the robotic device passively plantarflexed the subject’s ankle at two different velocities (reference velocity, Vr, and comparison velocity, Vc). After experiencing both movements, participants verbally indicated which one was perceived as faster (“first” or “second”)

As shown in Fig. 2A, each subject was seated on a chair with barefoot. The affected foot was secured to a foot pedal, and their eyes and ears were covered to eliminate visual and auditory interference. During testing, the researcher instructed the participants to relax and avoid voluntary contractions. The distance and height of the lateral malleolus relative to the heel were measured to align the axis of rotation of the ankle joint with the center of rotation of the apparatus actuator.

The testing protocol was controlled using a custom MATLAB program, which determined the order of stimulus presentation and the velocity differences required [24]. The testing procedure is presented in Fig. 2B. Each session consisted of 30 trials. The subjects were allowed to have three practice trials before the formal assessment to ensure that they understood the procedure. For each trial, two passive range of motion plantarflexion movements were delivered at a reference velocity (Vr, 15°/s) and a comparison velocity (Vc, 8.5°/s–14.8°/s), with Vc always slower than Vr. Movements began at the neutral ankle position. The ankle robot first plantarflexed the ankle at one velocity (either Vr or Vc); then, after a 2-second pause, returned it to the initial position before delivering the second movement. The order of the two velocities (Vr or Vc) was randomized across trials.

To control for potential time-related confounding variables, the total duration of each velocity movement was matched within a trial, although absolute movement times varied between trials. Moreover, the experimental design ensured that the difference between the two angular displacements remained below 4°, which is less than the average ankle position sense discrimination threshold in healthy young adults (approximately 2.4°) [38]. Maintaining displacement differences below this threshold minimizes the likelihood that the subjects could rely on final position cues to judge velocity differences, thereby isolating genuine motion sense discrimination. At the end of each trial, the subjects verbally indicated which movement felt faster (“first” or “second”). Based on their response, the subsequent velocity difference was adaptively adjusted using a Bayesian Psi-marginal algorithm.

After testing was completed, a logistic Weibull function was fitted to the dataset for each subject, which included stimulus size difference (i.e., the difference between reference and comparison ankle velocity stimuli) and the corresponding correct response rates. Based on the fitted function, the stimulus size difference associated with a 75% correct response rate was defined as the JND (^◦^/s), which represents the minimum difference in velocity required to discriminate a comparison from the reference velocity. The IU (^◦^/s) was defined as the range of stimulus size differences corresponding to correct response probabilities between 60% and 90%, which provides an index of perceptual precision. EEG was also recorded concurrently during the proprioceptive assessment to capture cortical electrophysiological activity.

### Assessment tools for functional performance

The study consisted of three functional tasks to assess functional performance: the single-leg stance test (SLS), single-leg hop test (SLH), and single-leg lateral hop test (SLLH). Before the assessment, the subjects were instructed to remove their socks and any gear on their feet. Participants adapted to standing barefoot for 3 min. The same assessor conducted all pre- and post-intervention assessments to ensure consistency. However, assessor blinding was not implemented due to the non-randomized, sequential study design and the visible nature of the taping conditions.

The SLS was used to measure static balance. Each participant performed the SLS with eyes open (EO) and eyes closed (EC). For each condition, the subject stood on their affected leg with hands placed on the iliac crests and the contralateral leg positioned so that the foot was slightly away from the affected medial malleolus of the affected side and did not touch the ground. They maintained a stable head posture and focused their attention on a visual target at eye level in front of them to minimize eye movements, or kept their EC. The test was concluded upon hearing the cue “Stop” after 30 s, if the support leg moved, or if the suspended leg touched the ground. No practice trials were permitted, and a minimum rest period of 1 min was provided between trials. The time maintained was recorded.

The SLH was used to measure lower-extremity power and stability during landing. The subjects stood on their affected leg with the heel aligned to a starting line on the ground. Next, they hopped forward as far as possible, using their arms freely for momentum. A correct landing required maintaining balance on the affected leg for 3 s without touching the ground with the unaffected leg or moving the support foot. The test ended if significant pain occurred or the participant failed to land correctly. Hop distance was recorded as the SLH performance outcome.

**Fig. 3:**
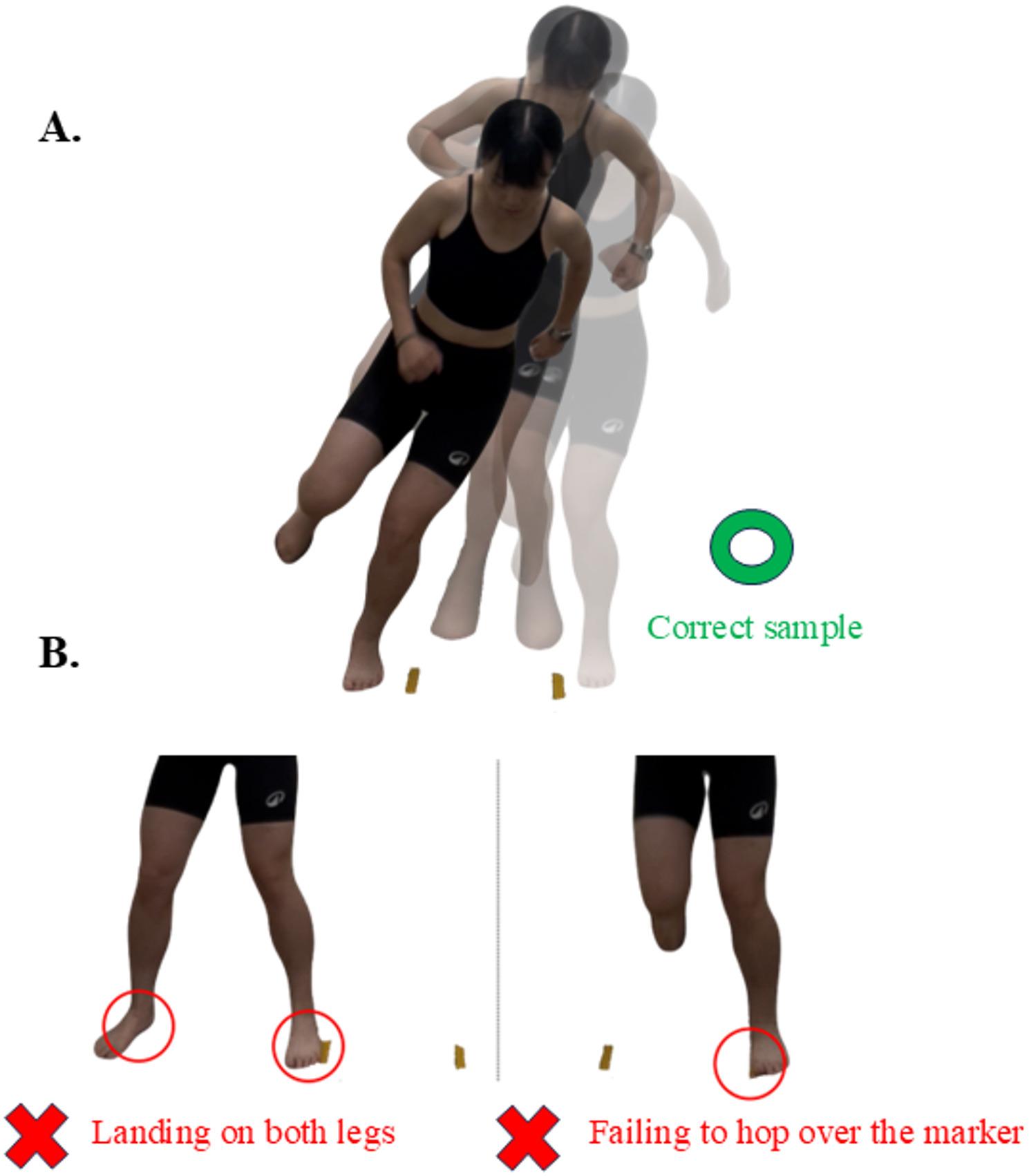
The single-leg lateral hop (SLLH) test. **A** Participants performed repeated lateral hops on their affected leg over two markers set at a distance equal to 22% of their height **B** An error was defined as failing to clear a marker or landing on both legs

The SLLH was used to measure dynamic balance, specifically in the frontal plane. As shown in Fig. 3A, participants hopped laterally over two markers, which were set apart at a distance equal to 22% of the participant’s height; participants hopped on their affected leg, as many times as possible within 30 s [39]. The test ended if significant pain or three consecutive errors (failing to hop over the marker or landing on both legs, as illustrated in Fig. 3B) occurred. The number of successful hops (SLLH-success) and errors (SLLH-error) was recorded.

### EEG recording and data processing

To capture cortical neural activity concurrent with ankle proprioceptive assessment, EEG activity was recorded using a Quik-Cap (Compumedics Neuroscan, El Paso, TX, USA) electrode cap with 32 scalp electrode arrays in accordance with the International 10–20 System. A ground electrode was positioned on the mid-forehead. EEG signals were filtered offline with a 0.1–50 Hz band-pass and referenced to linked mastoid electrodes. Using horizontal and vertical electrooculographic activities were monitored using ocular electrodes. Impedances were kept below 5 kΩ. The resulting data were digitized with a 60-Hz notch filter at an A/D rate of 500 Hz/channel using a Neuroscan SynAmps amplifier (Compumedics Neuroscan, El Paso, TX, USA).

During the ankle proprioceptive assessment, EEG activity was recorded simultaneously with the ankle motion apparatus to facilitate the time-synchronized ERP analysis. This enabled the simultaneous evaluation of psychophysical responses associated with ankle movement stimuli and their corresponding neural activity, which provided insight into the cortical mechanisms underlying proprioceptive processing. ERP data were extracted from the midline electrodes (Fz, FCz, and Cz) positioned over the sensorimotor cortex, which are responsible for processing lower-limb somatosensory input and evaluating motor-related stimuli. The electrodes were selected for their sensitivity to ERP components resulting from ankle proprioceptive-motor tasks [28, 29]. Continuous EEG data were segmented into epochs spanning from − 500 ms pre-stimulus onset to 1000 ms post-stimulus. The N1 component occurred within 100–200 ms after stimulus onset and was defined as the primary component of interest for analysis. To ensure signal quality, artifact rejection procedures were applied, which excluded epochs with amplitudes exceeding ± 100 µV.

### Statistical analysis

An a priori power analysis was performed using G*Power 3.1 for a repeated-measures ANOVA (within-subject design). Parameters were set to α = 0.05, power = 0.95, and an effect size of 0.33, based on the values reported by Yin and Wang [18], when evaluating the effects of KT on balance control. Accounting for a potential dropout rate of 15%, the minimum required sample size was estimated at 26 subjects.

All statistical analyses were performed using SPSS version 29.0.1.0 (IBM Corp., Armonk, NY, USA). Descriptive statistics were used to present the demographic and baseline characteristics of the subjects, including age, sex, height, weight, and CAIT questionnaire scores. Proprioception (JND, IU) and functional performance (SLS, SLH, SLLH) test results were each subjected to a two-way mixed design repeated-measures analysis of variance (RM-ANOVA) with 2 intervention conditions (35% tension vs. 0% tension) × 2 time points (pre-test vs. post-test). For the ERP results, N1 amplitude and latency were analyzed using a three-way RM-ANOVA with 2 intervention conditions (35% tension vs. 0% tension) × 2 time points (pre-test vs. post-test) × 3 electrode sites (Fz, FCz, and Cz). If significant effects were observed, Bonferroni’s adjusted pairwise comparisons were conducted. Normality and homogeneity were assessed using the Kolmogorov–Smirnov and Levene tests, respectively. The Greenhouse–Geisser correction was applied if the assumption of sphericity was violated. Statistical significance was set at *p* < 0.05, and partial eta-squared (*n*_*p*_²) was reported as the measure of effect size. Pearson correlation coefficients were calculated to determine the associations between changes in proprioception outcomes, functional performance outcomes, and ERP indices.

## Results

### Demographic characteristics

This study recruited 30 college athletes with CAI. Table 1 presents the descriptive data, including age, height, weight, and CAIT score. Of the subjects, 15 (50%) were male, and 9 (30%) had bilateral CAI. For those with bilateral involvement, the more affected side was defined as the ankle with the lower CAIT score. In addition, 16 participants (53.3%) had their dominant side as the affected ankle.

**Table 1:** Demographic characteristics of the subjects

	Mean (SD)	Min	Max
Age (years)	21.00 (2.35)	18	25
Height (cm)	170.53 (9.56)	145	185
Weight (kg)	65.20 (11.28)	49	90
CAIT score	17.17 (4.36)	7	24

*CAIT* Cumberland Ankle Instability Tool

### Proprioception

**Fig. 4:**
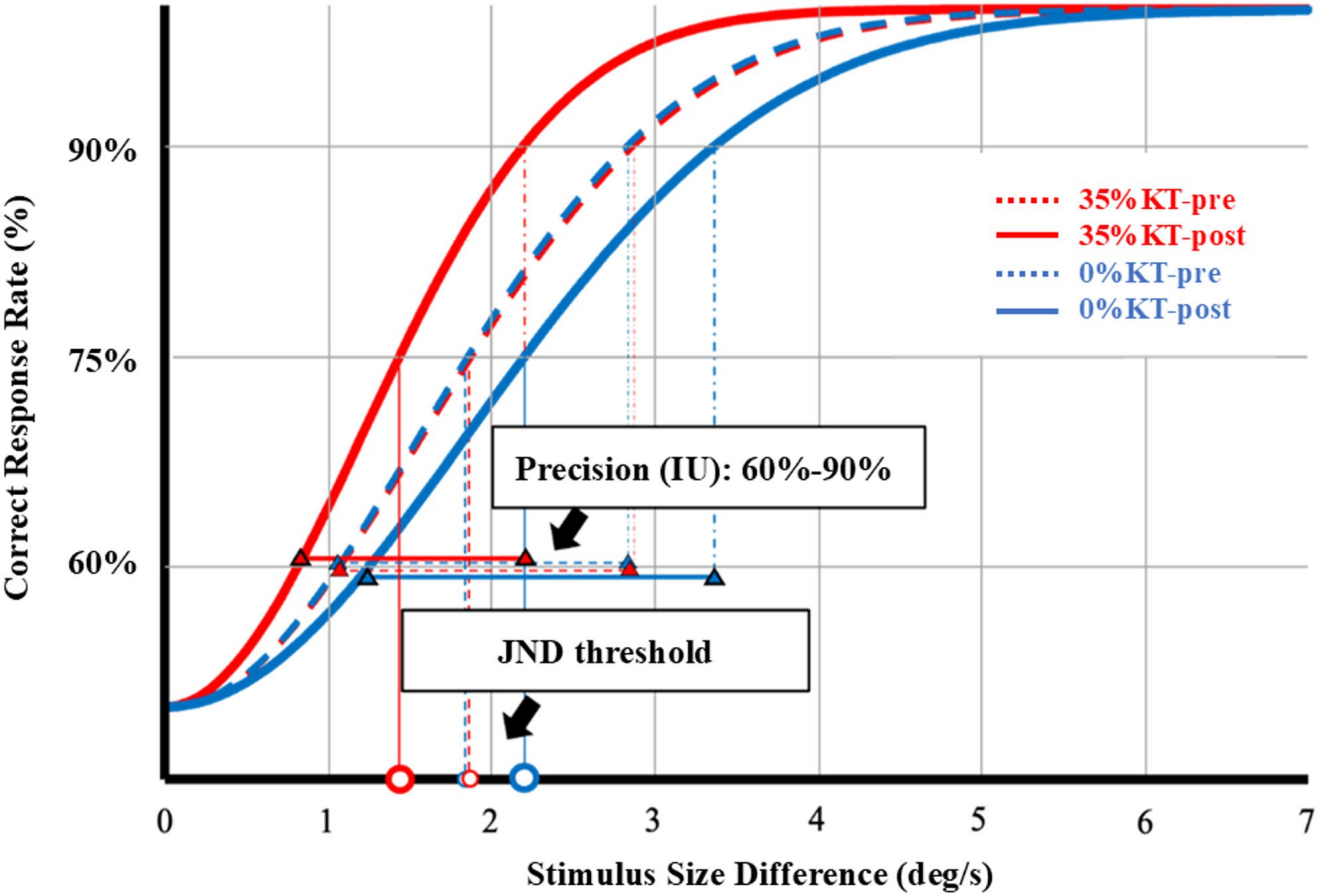
Example of derived stimulus–response psychometric functions for each condition at pre- and post-test. The open circle marks the just noticeable difference (JND), defined as the stimulus size difference corresponding to a 75% correct response rate. The interval of uncertainty (IU) is shown by a double-headed line with triangle endpoints, representing the range of stimulus size differences corresponding to correct response probabilities between 60% and 90%

As illustrated in Fig. 4; Table 2, the RM-ANOVA on JND revealed a significant *time × condition* interaction[*F*(1,58) = 15.15, *p* < 0.001, *n*_*p*_^*2*^= 0.21]. *Post hoc* analysis revealed that JND significantly decreased following 35% tension KT application (pre vs. post: 1.86 ± 0.83 vs. 1.43 ± 0.53, *p* = 0.004). Conversely, JND significantly increased following 0% tension KT application (pre vs. post: 1.83 ± 0.62 ^◦^/s vs. 2.19 ± 0.76 ^◦^/s, *p* = 0.015). Moreover, for post-KT assessment, the JND was significantly higher in the 0% tension KT condition compared with that under the 35% tension KT condition (2.19 ± 0.76 ^◦^/s vs. 1.43 ± 0.53 ^◦^/s, *p* < 0.001). A significant main effect of *condition* was also observed [*F*(1,58) = 6.10, *p* = 0.017, *n*_*p*_^*2*^= 0.10], indicating that JND was overall greater under the 0% tension KT condition (2.01 ± 0.69 ^◦^/s) than under the 35% tension KT condition (1.65 ± 0.68 ^◦^/s). No significant main effect of time was detected.

**Table 2:** Statistical results for Proprioception, functional Performance, and electrophysiological measures

	Interaction Effect	Main effect	Post hoc findings
**Proprioception**			
JND (^◦^/s)	Time × Condition* [*F*(1,58) = 15.15, *p* < 0.001, *n*_*p*_^*2*^= 0.21]	*Time* [*F*(1,58) = 0.12, *p* = 0.728, *n*_*p*_^*2*^= 0.00] Condition* [*F*(1,58) = 6.10, *p* = 0.017, *n*_*p*_^*2*^= 0.10]	↓ in 35% tension KT ↑ in 0% tension KT Post KT: 0% tension KT > 35% tension KT
IU (^◦^/s)	*Time × Condition* [*F*(1,58) = 3.79, *p* = 0.057, *n*_*p*_^*2*^= 0.06]	*Time* [*F*(1,58) = 0.00, *p* = 0.959, *n*_*p*_^*2*^= 0.00] *Condition* [*F*(1,58) = 1.79, *p* = 0.186, *n*_*p*_^*2*^= 0.03]	n/a
Functional performance			
SLS-EO (s)	*Time × Condition* [*F*(1,58) = 0.10, *p* = 0.748, *n*_*p*_^*2*^ = 0.00]	*Time* [*F*(1,58) = 3.70, *p* = 0.059, *n*_*p*_^*2*^= 0.06] *Condition* [*F*(1,58) = 0.55, *p* = 0.463, *n*_*p*_^*2*^= 0.01]	ns
SLS-EC(s)	Time × Condition* [*F*(1,58) = 8.09, *p* = 0.006, *n*_*p*_^*2*^ = 0.12]	*Time* [*F*(1,58) = 3.69, *p* = 0.060, *n*_*p*_^*2*^= 0.06] *Condition* [*F*(1,58) = 0.00, *p* = 0.990, *n*_*p*_^*2*^= 0.00]	↑ in 35% tension KT
SLH (cm)	*Time × Condition* [*F*(1,58) = 1.34, *p* = 0.253, *n*_*p*_^*2*^ = 0.02]	*Time* [*F*(1,58) = 2.83, *p* = 0.098, *n*_*p*_^*2*^ = 0.05] *Condition* [*F*(1,58) = 0.07, *p* = 0.789, *n*_*p*_^*2*^= 0.00]	ns
SLLH-success (count)	Time × Condition* [*F*(1,58) = 7.84, *p* = 0.007, *n*_*p*_^*2*^ = 0.12]	Time* [F(1,58) = 33.94, *p* < 0.001, *n*_*p*_^*2*^ = 0.37] *Condition* [*F*(1,58) = 2.16, *p* = 0.147, *n*_*p*_^*2*^= 0.04]	Post KT: 35% tension KT > 0% tension KT
SLLH-error (count)	Time × Condition* [*F*(1,58) = 5.77, *p* = 0.020, *n*_*p*_^*2*^ = 0.09]	*Time* [F(1,58) = 0.45, *p* < 0.504, *n*_*p*_^*2*^ = 0.01] Condition* [*F*(1,58) = 6.28, *p* = 0.015, *n*_*p*_^*2*^= 0.10]	↑ in 0% tension KT Post KT: 0% tension KT > 35% tension KT
**Electrophysiology**			
N1 amplitude	Time × Condition* [*F*(1, 58) = 8.95, *p* = 0.004, *n*_*p*_^*2*^= 0.13] *Electrode× Condition* [*F*(2, 57) = 1.54, *p* = 0.223, *n*_*p*_^*2*^= 0.05] *Time × Electrode* [*F*(2, 57) = 1.67, *p* = 0.197, *n*_*p*_^*2*^= 0.06] *Time × Electrode × Condition* [*F*(2, 57) = 1.65, *p* = 0.202, *n*_*p*_^*2*^= 0.06]	*Time* [*F*(1, 58) = 1.19, *p* = 0.281, *n*_*p*_^*2*^ = 0.02] *Condition* [*F*(1, 58) = 0.25, *p* = 0.619, *n*_*p*_^*2*^ = 0.00] Electrode* [*F*(2, 57) = 9.367, *p* < 0.001, *n*_*p*_^*2*^ = 0.25]	↓ in 0% tension KT
N1 latency	*Time × Condition* [*F*(1, 58) = 1.25, *p* = 0.269, *n*_*p*_^*2*^= 0.02] *Electrode× Condition* [*F*(2, 57) = 0.30, *p* = 0.739, *n*_*p*_^*2*^= 0.01] *Time × Electrode* [*F*(2, 57) = 0.14, *p* = 0.867, *n*_*p*_^*2*^= 0.01] *Time × Electrode × Condition* [*F*(2, 57) = 1.64, *p* = 0.203, *n*_*p*_^*2*^= 0.05]	*Time* [*F*(1, 58) = 0.98, *p* = 0.327, *n*_*p*_^*2*^= 0.02] *Condition* [*F*(1, 58) = 0.69, *p* = 0.410, *n*_*p*_^*2*^ = 0.01] *Electrode* [*F*(2, 57) = 1.41, *p* = 0.252, *n*_*p*_^*2*^ = 0.05]	ns

*JND* Just noticeable difference, *IU* Interval of uncertainty, *SLS-EO* Single-leg stance with eyes open, *SLS-EC* Single-leg stance with eyes closed, *SLH* single-leg hop, *SLLH* single-leg lateral hop, *ns* Non-significant, *KT* Kinesiology tape

*p < 0.05, significant interaction or main effect

Regarding IU, RM-ANOVA showed no significant interaction or main effects of *time*,* condition*,* or group.*

### Functional performance

The RM-ANOVA on the SLS-EO revealed no significant main effects or interaction effects associated with *time*,* condition*,* or group.* For SLS-EC, a significant *time × condition* interaction was evident [*F*(1,58) = 8.09, *p* = 0.006, *n*_*p*_^*2*^ = 0.12]. *Post hoc* analysis revealed that the SLS-EC time significantly increased following 35% tension KT application (pre vs. post: 16.86 ± 10.50 s vs. 21.73 ± 9.46 s, *p* = 0.001).

The RM-ANOVA on the SLH showed no significant main effects associated with *group*,* time*,* condition*, or any significant interactions between these factors.

For SLLH-success, a significant time × condition interaction was observed [*F*(1,58) = 7.84, *p* = 0.007, *n*_*p*_^*2*^ = 0.12]. *Post hoc* analysis revealed higher post-KT SLLH-success scores in the 35% tension KT condition compared with the 0% tension KT condition (37.10 ± 4.89 vs. 33.57 ± 7.35, *p* = 0.032). A significant main effect of time was also found [*F*(1,58) = 33.94, *p* < 0.001, *n*_*p*_^*2*^ = 0.37], indicating overall improvement in SLLH-success from pre- to post-KT (32.77 ± 6.31 vs. 35.33 ± 6.44).

For SLLH-error, the RM-ANOVA revealed a significant *time x condition* interaction effect [*F*(1,58) = 5.77, *p* = 0.020, *n*_*p*_^*2*^= 0.09]. Post hoc analysis indicated that errors increased significantly under the 0% tension KT conditions post-KT (pre vs. post: 3.37 ± 2.61 vs. 4.43 ± 4.05, *p* = 0.034), and post-KT errors were significantly higher in the 0% tension KT condition compared with the 35% tension KT condition (4.43 ± 4.05 vs. 1.97 ± 2.40, *p* = 0.006). In addition, a significant main effect of condition was found [F(1,58) = 6.28, *p* = 0.015, *n*_*p*_^*2*^ = 0.10], with overall higher error counts observed in the 0% tension KT condition (3.90 ± 3.3) compared with the 35% tension KT condition (2.27 ± 2.17).

### Electrophysiological indices

**Fig. 5:**
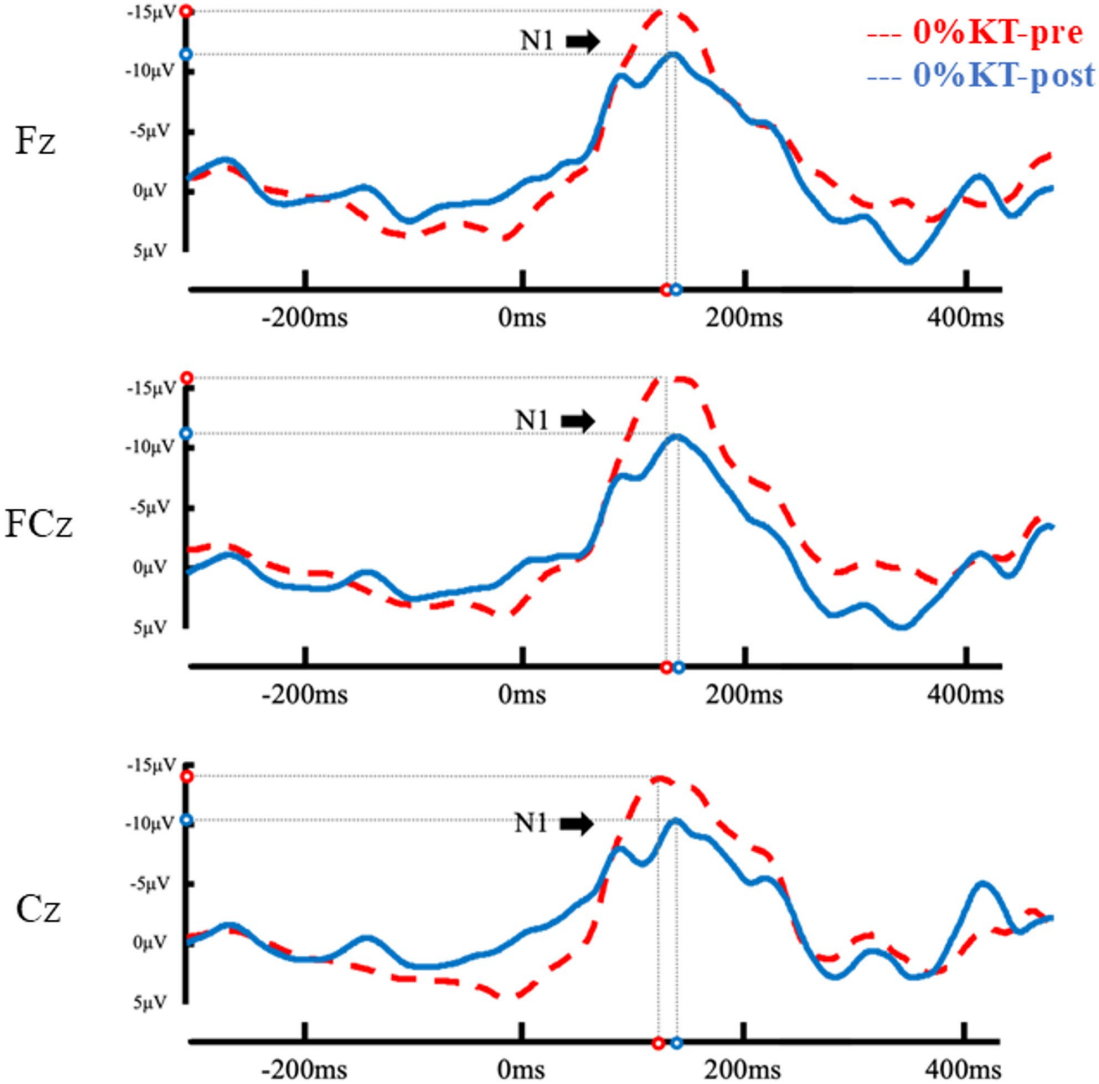
Event-related potential (ERP) waveforms under the 0% tension KT condition at pre- and post-test. Grand-averaged traces are shown at midline electrodes. Open circles indicate the N1 peak amplitude and latency

**Fig. 6:**
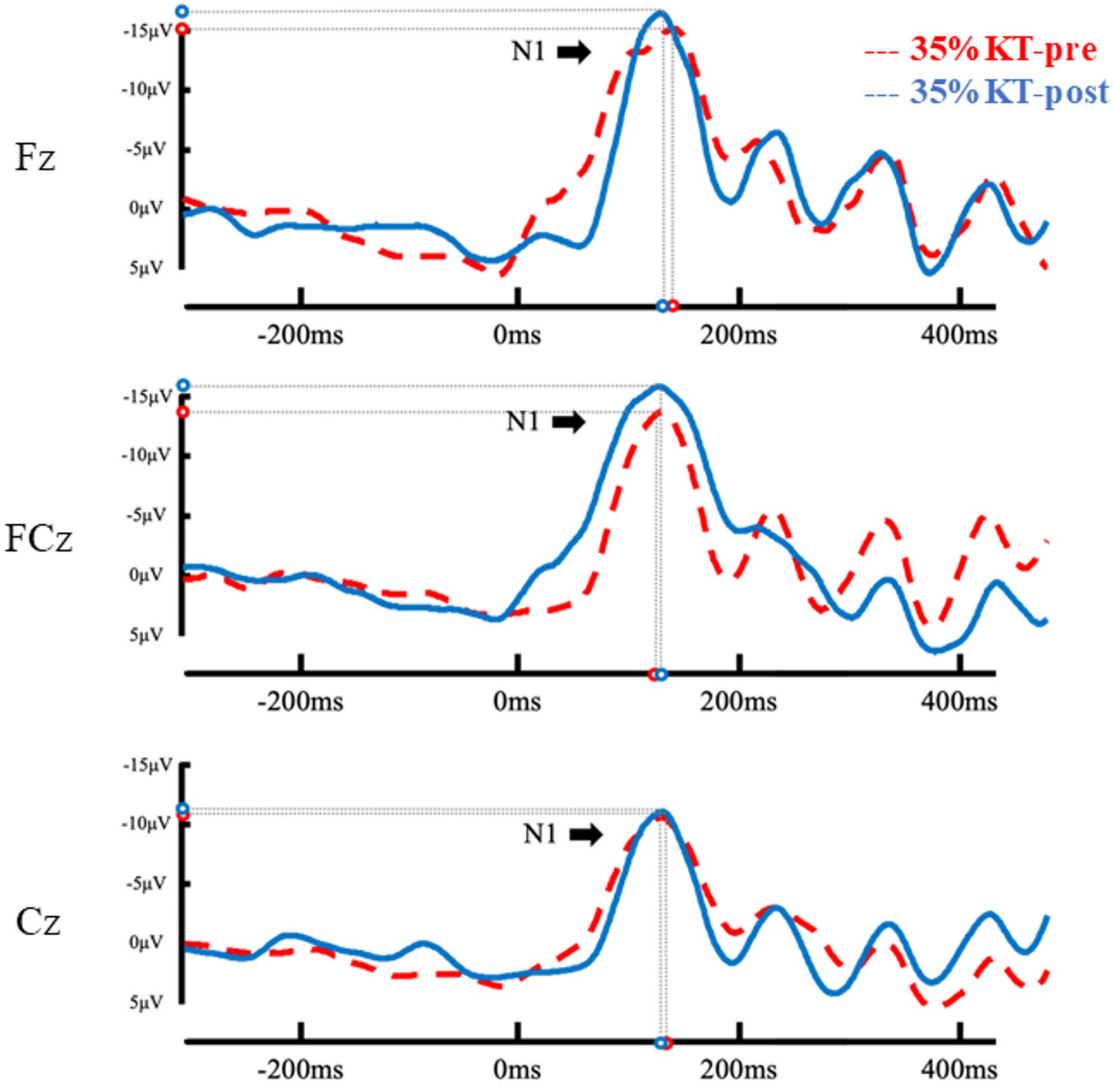
Event-related potential (ERP) waveforms under the 35% tension KT condition at pre- and post-test. Grand-averaged traces are shown at midline electrodes. Open circles indicate the N1 peak amplitude and latency

As shown in Figs. 5 and 6; Table 2, the RM-ANOVA revealed a significant time × condition interaction on N1 amplitude [*F*(1, 58) = 8.95, *p* = 0.004, *n*_*p*_^*2*^= 0.13]. *Post hoc* comparisons showed that a significant reduction in N1 amplitude was observed following 0% tension KT at post-test (pre: −15.23 ± 7.13 µV; post: −11.91 ± 5.75 µV; *p* = 0.005). In contrast, N1 amplitude did not change significantly from pre- to post-test in the 35% tension KT condition (pre: −13.64 ± 8.67 µV; post: −15.18 ± 10.42 µV; *p* = 0.184). A significant main effect of electrode was also observed [*F*(2, 57) = 9.367, *p* < 0.001, *n*_*p*_^*2*^ = 0.25]. Pairwise comparisons indicated that amplitudes at Fz (− 15.21 ± 7.36 µV) were significantly larger (more negative) than at Cz (− 12.23 ± 7.52 µV) (mean difference = 2.98 µV, *p* < 0.001). FCz (− 14.54 ± 9.11 µV) was likewise significantly larger than Cz (mean difference = 2.31 µV, *p* = 0.006). No significant difference was found between Fz and FCz. All other main effects and interactions for N1 amplitude and latency were not statistically significant.

### Correlations

For the correlation analyses, change scores ($\:\varDelta\:$) were calculated for each dependent variable as the difference between post- and pre-test values ($\:\varDelta\:$X = X_post_ – X_pre_). These scores were used to evaluate the relationships between the proprioceptive measures (JND, IU), electrophysiological indices (N1 amplitude, N1 latency), and functional performance outcomes (SLS, SLH, SLLH).

Across time points and conditions, $\:\varDelta\:$JND was significantly correlated with $\:\varDelta\:$N1 amplitude at Fz (*r* = 0.45, *p* < 0.001), FCz (*r* = 0.41, *p* = 0.001), and Cz (*r* = 0.27, *p* = 0.039), as well as with $\:\varDelta\:$N1 latency at Cz (*r* = 0.29, *p* = 0.027) (Table 3). No significant correlations were observed between the $\:\varDelta\:$IU and $\:\varDelta\:$N1 components. In addition, $\:\varDelta\:$JND was significantly correlated with $\:\varDelta\:$SLLH-success (*r* = $\:-\:$0.45, *p* < 0.001) and $\:\varDelta\:$SLLH-error (*r* = 0.46, *p* < 0.001). Similarly, $\:\varDelta\:$IU was significantly correlated with $\:\varDelta\:$SLLH-success (*r* =$\:-\:$0.39, *p* = 0.002) and $\:\varDelta\:$SLLH-error (*r* = 0.30, *p* = 0.019).

**Table 3:** Correlations among changes in proprioception, electrophysiological indices, and functional performance

Variable Pair	Correlation coefficient (*r*)	*p* value
∆JND – ∆N1 amplitude (Fz)	0.45	< 0.001
∆JND – ∆N1 amplitude (FCz)	0.41	0.001
∆JND – ∆N1 amplitude (Cz)	0.27	0.039
∆JND – ∆N1 latency (Cz)	0.29	0.027
∆JND – ∆SLLH-success	−0.45	< 0.001
∆JND – ∆SLLH-error	0.46	< 0.001
∆IU – ∆SLLH-success	−0.39	0.002
∆IU – ∆SLLH-error	0.30	0.019

∆:= post–pre change score; JND: Just noticeable difference; IU: Interval of uncertainty; SLLH: single-leg lateral hop

Only significant correlations are presented

## Discussion

### Main finding

This study is the first to examine the effects of different KT tensions on proprioception and functional performance in individuals with CAI using psychophysical, functional and electrophysiological measurements. Applying 35% tension KT improved proprioceptive acuity, evidenced by reduced JND, while 0% tension KT increased JND. Functional outcomes mirrored these effects, with better SLS-EC and higher SLLH-success under 35% tension KT, whereas the SLLH-error increased following 0% tension KT. At the cortical level, 0% tension KT reduced N1 amplitude. Correlational analyses further linked proprioceptive measures with N1 parameters and SLLH performance, underscoring the interplay between sensory acuity, cortical responsiveness, and motor stability.

### Proprioception

JND and IU are two representative indices that reflect an individual’s proprioception. Consistent with our hypothesis, JND was significantly decreased following 35% tension KT, indicating an immediate positive effect of KT on enhancing motion-detecting ability. This is consistent with previous studies on knee joint position sense, which indicate that appropriate tension (15–40%) with specific directional taping (from origin to insertion) yields beneficial effects on proprioception [36]. The underlying mechanism may be explained by sensory physiology, in which proprioceptive input is mediated by muscle spindles, Golgi tendon organs, joint receptors, and cutaneous mechanoreceptors, particularly Ruffini and Pacinian corpuscles [40]. Ruffini corpuscles respond slowly to sustained pressure and skin stretch, whereas Pacinian corpuscles are rapidly adapting and respond to vibration and sudden disturbances [41]. Signals from these receptors ascend through the dorsal column–medial lemniscus pathway to the somatosensory cortex and through the spinocerebellar tract for motor regulation [29]. Application of KT with appropriate tension induces localized traction and displacement of the skin, which effectively stimulates mechanoreceptors and strengthens sensory input.

Interestingly, JND increased following 0% tension KT, suggesting that motion sense acuity was disrupted rather than enhanced. This is consistent with the results of Justo-Cousiño et al. [42], who reported poorer wrist proprioception under 0% tension KT, and with Ghai et al. [43], who found that placebo taping impairs proprioceptive performance. One possible explanation is that KT without tension generates inconsistent or ambiguous cutaneous input. Instead of enhancing mechanoreceptor activity through skin stretch, 0% tension KT may induce irregular sensory signals or even introduce sensory noise that disrupts central integration. Moreover, individuals may subconsciously anticipate supportive feedback from KT. When tape is applied with no tension, these expected cues are absent, which results in a mismatch between anticipated and actual sensory input. This sensory incongruence may disrupt sensorimotor processing and increase perceptual uncertainty. Therefore, placebo KT should not be regarded as neutral. It may actively interfere with mechanoreceptive signaling and proprioceptive function when applied without adequate tension.

IU showed no changes across taping conditions, which was in contrast to the significant effects observed for JND. This indicates that KT may influence proprioceptive sensitivity, but not perceptual precision or consistency. IU is indicative of the reliability and stability of proprioceptive judgments among the trials [24], which may depend on broader neural processes beyond cutaneous input, including multisensory integration or higher-order decision mechanisms. Moreover, maintaining perceptual reliability may require repeated or sustained proprioceptive stimulation to induce cortical recalibration. These processes may not be sufficiently enhanced by a single KT intervention, which may explain why IU, which reflects perceptual reliability, was unchanged in the present study. Future studies should determine whether IU requires longer-term adaptation or repeated exposures to taping before measurable improvements emerge.

### Functional performance indices

In the present study, the performance of SLS-EO was not affected by KT intervention. This may be attributed to a ceiling effect, in which most participants were able to maintain a position for nearly 30 s at baseline. This suggests that the task was not sufficiently challenging for these athletes. Under such circumstances, meaningful improvements were difficult to detect. In contrast, a significant effect emerged under SLS-EC conditions, in which 35% tension KT enhanced balance performance. Song et al. [44] reported that individuals with CAI tend to rely heavily on visual input during SLS tasks. When visual input was deprived during eyes-closed conditions, the subjects were forced to rely on somatosensory information. In this challenging scenario, the facilitation of proprioceptive input provided by 35% tension KT was evident; however, our results are inconsistent with those of Yin and Wang [18], who reported no significant differences in postural sway between KT, placebo taping, or no taping during the Sensory Organization Test (SOT), even with visual deprivation. Notably, their taping method resembled rigid AT rather than KT, and did not align with muscle orientation. It also used a 10-second balance task, which differs from the 30-second protocol used in the present study. Similarly, de-la-Torre-Domingo et al. [45] found that both KT and placebo taping improved SOT composite scores (KT: 80.47 ± 3.90 to 83.87 ± 2.97; placebo: 80.20 ± 5.65 to 84.33 ± 4.15; *p* = 0.726), suggesting that balance enhancement may stem from subjective stability rather than tape tension-specific sensory input. Moreover, Lee et al. [46] reported that KT applied with 0–10% tension only reduced sway area during a 15-second SLS test, but had limited effects on sway velocity and distance, which indicates that low-tension KT only has a modest effect on balance outcomes. Taken together, the effect of KT on SLS likely depends on factors such as tape tension, taping technique, outcome measures, participant characteristics, and task difficulty. In the present study, the improvement observed in SLS-EC may be attributed to the use of higher tension, muscle-oriented applications, and a more challenging balance condition. Overall, 35% tension KT may provide sufficient cutaneous shear force to attenuate proprioceptive input when proprioceptive demand is high, thus enhancing balance performance in athletes with CAI.

In the SLH task, no significant changes in hop distance were observed with either KT condition, suggesting that its application did not further enhance performance in movements that were primarily dependent on muscular strength and explosive power. This is consistent with the results of previous studies. For example, de Jesus et al. [41] applied KT to the quadriceps of healthy adults over a range of tensions (0%–100%) and time points (immediately, 3 days, 5 days, and 72 h after removal) and observed no significant effects on hop distance. Similarly, Poon et al. [47] found that neither 35% tension KT nor placebo KT (0% tension) improved peak torque, total work, or time to peak torque during isokinetic knee extension and flexion at 60°/s and 180°/s using a calibrated dynamometer. In studies involving athletes with CAI, Bicici et al. [15] also demonstrated that KT applied to the ankle did not enhance vertical jump or hop performance. Instead, it preserved the baseline performance, which contrasts with rigid AT, which restricted plantarflexion and significantly reduced jump outcomes. Taken together, these results suggest that while KT may enhance sensory input, explosive performance in dynamic tasks such as SLH primarily depends on the intrinsic mechanical and musculotendinous properties of the lower limbs and is not substantially enhanced by KT application [48].

Unlike the previous tasks, the SLLH test relies heavily on dynamic stability in the frontal plane, particularly in the inversion–eversion direction, which is affected by CAI deficits. Thus, the SLLH test is particularly sensitive for capturing meaningful changes in motor control. In this task, when participants were administered 35% tension KT, they demonstrated clear improvements in hop quality, with fewer errors and more successful landings during the post-test. These results indicate that KT with appropriate tension enhances functional control during repetitive lateral movements. Supporting evidence from Bicici et al. [15] indicated that 25% tension KT tension facilitates successful performance in single-leg hurdle hop tasks, whereas Wang et al. [23] reported that 20–35% tension KT reduces ankle inversion angles during side-step cutting movements, thereby improving kinematic alignment and stability.

In contrast to the improvements observed with 35% tension KT, the 0% tension conditions resulted in more landing errors, which suggests that placebo taping may impair functional performance rather than remain neutral. Similar to the results for JND, KT without sufficient tension provides ambiguous cutaneous input, failing to engage mechanoreceptors effectively. This generates irregular afferent signals and disrupts sensorimotor integration. Moreover, KT can provide athletes with a subjective sense of stability and confidence, which encourages them to push their performance beyond their natural control capacity [45]. However, when KT is applied with inappropriate tension, the distorted sensory input conflicts with the perception of support, which results in a mismatch between expected and actual feedback. This discrepancy compromises neuromuscular control, increases the likelihood of technical errors, such as faulty landings, and ultimately increases the risk of ankle injury. Taken together, these results indicate that the benefits of KT primarily depend on applying the appropriate tension, which ensures both accurate proprioceptive feedback and safe functional performance.

### Electrophysiological indices

In the proprioception test with simultaneous ERP analysis, N1 amplitude was stable under 35% tension KT conditions, but decreased significantly under 0% tension KT conditions. This indicates that appropriate KT tension preserves proprioceptive processing and cortical responsiveness, whereas 0% tension may suppress sensory input and reduce early cortical activation. Previous studies have indicated that smaller N1 amplitudes are linked to poorer proprioceptive performance [28, 29], a pattern also reflected in the present study with larger JND values and smaller N1 amplitudes under 0% tension KT conditions. In contrast, N1 latency was not different between the two conditions, suggesting that KT attenuates the strength of cortical responses rather than the speed of signal transmission. This is consistent with evidence that N1 latency is relatively stable and typically altered in cases of neural degeneration, such as aging [29]. Taken together, these results suggest that KT influences proprioceptive function at sensory and cortical processing levels, with N1 amplitude serving as a sensitive marker of these changes.

Although ERP studies specifically targeting ankle proprioception are limited, the results from other joints provide mechanistic insight into the origins of N1. Mima et al. [49] demonstrated that muscle spindles are the primary afferent source required for generating N1, whereas cutaneous and joint mechanoreceptors modulate its amplitude and timing. When applying this framework to our results, 35% tension KT may have enhanced cutaneous stimulation through skin and fascia traction, thereby strengthening afferent input and maintaining robust cortical responses. In contrast, 0% tension KT may have disrupted normal receptor signaling, resulting in decreased N1 amplitudes similar to those observed under conditions of cutaneous receptor blockade. Collectively, these findings suggest that the beneficial effects of KT arise not only from mechanical support but also from optimized sensory input that facilitates cortical proprioceptive processing. Conversely, improperly applied KT, such as 0% tension taping, could act as a disruptive stimulus, diminishing both peripheral and cortical aspects of proprioceptive function.

### Correlation analysis

In the present study, the correlation results provide additional evidence linking proprioceptive acuity, cortical processing, and functional performance. $\:\varDelta\:$JND was positively correlated with $\:\varDelta\:$N1 amplitude across the frontal and central electrodes (Fz, FCz, Cz), and with $\:\varDelta\:$N1 latency at Cz. This indicates that when proprioceptive acuity deteriorates (larger JND), cortical responses weaken (N1 becomes less negative in amplitude) and slow (longer latency). Conversely, improvements in proprioceptive acuity are accompanied by more robust and efficient cortical processing of sensory input. This reinforces the role of N1 amplitude as a sensitive neural marker for proprioceptive integrity. In contrast, $\:\varDelta\:$IU showed no significant correlation with the ERP indices, indicating that perceptual precision may depend on neural processes beyond those captured by early cortical activity. Importantly, $\:\varDelta\:$JND and $\:\varDelta\:$IU exhibited significant positive correlations with $\:\varDelta\:$SLLH-success and negative correlations with $\:\varDelta\:$SLLH-errors. These results indicate that proprioceptive improvements translate to better movement accuracy and stability.

### Strengths and weaknesses

A major strength of our study is that it is the first to examine the effects of KT on proprioception, functional performance, and neurophysiological mechanisms in individuals with CAI. By integrating psychophysical measures, functional performance outcomes, and electrophysiological indices, we provide a multidimensional perspective on how KT modulates sensorimotor control. The inclusion of ERP analysis, particularly the N1 component, is also novel, as it offers mechanistic insight into cortical processing of proprioceptive input. Moreover, the study revealed distinct effects of different KT tensions, which are relevant to clinical applications and evidence-based taping strategies.

Several limitations should be noted. First, the relatively small sample size may limit statistical power and generalizability to broader athletic or clinical populations. Second, only the immediate effects of KT were evaluated. Thus, it remains unclear whether the observed benefits persist over time or with repeated application. Third, we only evaluated one taping technique and two levels of tension (0% and 35%). Other taping directions, applications targeting different muscle groups, or alternative levels of tension may yield different results, which limits the ability to generalize our results to other KT practices. Fourth, although proprioceptive retention is generally reported to last only five to ten days [33], the interval between the two KT conditions in this study was considered adequate. Nonetheless, some degree of carryover cannot be fully ruled out. It is worth noting, however, that the present within-subject design helped reduce variability stemming from individual differences, which might otherwise obscure the true effects of the intervention [50]. Future studies with larger, more diverse samples, randomized condition orders, and long-term follow-up are warranted to validate and extend these findings.

## Conclusion

In the present study, 35% tension KT significantly reduced JND, reflecting enhanced motion sense acuity, whereas IU remained unchanged, suggesting that proprioceptive reliability was not immediately affected. Conversely, 0% tension KT increased JND, indicating disruption of proprioceptive sensitivity. Functional performance outcomes paralleled these findings, with improvements in balance and lateral hop tasks under 35% tension KT conditions and increased landing errors under 0% tension KT conditions. ERP analysis showed that N1 amplitude remained stable under 35% tension KT conditions, but decreased under 0% tension KT conditions, which is consistent with poorer proprioceptive performance. Correlation analysis showed that changes in JND were linked to N1 responses and SLLH performance, indicating that proprioceptive improvements are closely coupled with cortical processing and functional stability. Overall, these results demonstrate that KT with appropriate tension facilitates both peripheral and cortical proprioceptive processing, thereby enhancing sensorimotor performance in athletes with CAI, while tensionless KT may introduce maladaptive sensory noise that compromises control. From a practical perspective, applying KT at 35% tension may support proprioceptive retraining and injury prevention strategies in rehabilitation or athletic settings.

## Data Availability

The datasets used and/or analyzed during the current study are available from the corresponding author on reasonable request.

## Abbreviations

CAI: Chronic ankle instability
KT: Kinesiology taping
JND: Just noticeable difference
IU: Interval of uncertainty
EEG: Electroencephalography
ERPs: Event-related potentials
SLS: Single-leg stance
SLLH: Single-leg lateral hop
TTDPM: Threshold for detection of passive motion
